# Supplementary material for: Reducing the Cost of Neural Network Potential Generation for Reactive Molecular Systems
Source: J Chem Theory Comput. 2023 Sep 25;19(19):6589–604. doi: 10.1021/acs.jctc.3c00391 (PMC10569056; doi:10.1021/acs.jctc.3c00391)
Supplement: Supplementary file 1 — ct3c00391_si_001.pdf [file ct3c00391_si_001.pdf]

Supporting information for: Reducing the cost of neural network potential generation for reactive molecular systems

Krystof Brezina, Hubert Beck, and Ondrej Marsalek[a)]
Charles University, Faculty of Mathematics and Physics, Ke Karlovu 3, 121 16 Prague 2, Czech Republic

(Dated: 25 July 2023)

S1. ADDITIONAL THEORY DETAILS

The following section contains additional details on the theory discussed in the main text. First, the derivation of the effective quantum temperature of the quantum harmonic oscillator is presented. This is followed by the discussion of the density-matching condition needed in the TTS procedure for appending thermal NMS of the minima away from MEPs.

Quantum effective temperature

To derive the expression for the quantum effective temperature, we start from the standard Hamiltonian for a 1D harmonic oscillator in a mass-weighted coordinate Ω with the natural frequency ω

$$\widehat{H} = -\frac{\hbar^2}{2}\frac{\mathrm{d}^2}{\mathrm{d}\Omega^2} + \frac{1}{2}\omega^2\Omega^2. \tag{S1}$$

The n-th solution for the corresponding time-independent Schrödinger equation ($n = 0, 1, 2, \ldots, \infty$) is in the well-known form based on the physicist's Hermite polynomials H_n

$$\psi_n(\Omega) = \left(\frac{1}{2^n n!\sqrt{\pi}}\right)^{\frac{1}{2}} \left(\frac{\omega}{\hbar}\right)^{\frac{1}{4}} e^{-\frac{\omega\Omega^2}{2\hbar}} H_n\left(\sqrt{\frac{\omega}{\hbar}}\Omega\right), \tag{S2}$$

which can be simplified to

$$\psi_n(y) = \left(\frac{\alpha}{2^n n!\sqrt{\pi}}\right)^{\frac{1}{2}} e^{-\frac{y^2}{2}} H_n(y) \tag{S3}$$

with the substitutions $\alpha = \sqrt{\frac{\omega}{\hbar}}$ and $y = \alpha\Omega$. The corresponding energy levels are

$$E_n = \hbar\omega\left(n + \frac{1}{2}\right). \tag{S4}$$

Ultimately, we are interested in the thermal density of the harmonic oscillator $\rho(y)$, which is formally obtained as the diagonal elements of the full density matrix $\rho(y, y')$. A formula for the density matrix in terms of an infinite sum over all states can be obtained as

$$\begin{aligned}\rho(y, y') &= \langle y'| e^{-\beta\widehat{H}} |y\rangle \\ &= \sum_{n,m} \langle y'|\psi_n\rangle \langle\psi_n| e^{-\beta\widehat{H}} |\psi_m\rangle \langle\psi_m|y\rangle \\ &= \sum_n \psi_n^*(y')\psi_n(y)e^{-\beta E_n} \\ &= e^{-\frac{\beta\hbar\omega}{2}} \frac{\alpha}{\sqrt{\pi}} \sum_n \frac{1}{2^n n!} e^{-\beta\hbar\omega n} \cdot \\ &\cdot e^{-\frac{y^2+y'^2}{2}} H_n(y)H_n(y'),\end{aligned} \tag{S5}$$

which can be closed using the so-called Mehler kernel[S1]

$$\sum_n \frac{\chi^n}{2^n n!} H_n(x)H_n(y) = \frac{1}{\sqrt{1-\chi^2}} e^{-\frac{\chi^2(x^2+y^2)-2\chi xy}{1-\chi^2}} : \tag{S6}$$

a mathematical identity for a parameter χ that allows summing over the above product of Hermite polynomials. With this, one obtains

$$\begin{aligned}\rho(y, y') &= e^{-\frac{\beta\hbar\omega}{2}} \frac{\alpha}{\sqrt{\pi}} e^{-\frac{y^2+y'^2}{2}} \frac{1}{\sqrt{1-e^{-2\beta\hbar\omega}}} \cdot \\ &\cdot e^{-\frac{e^{-2\beta\hbar\omega}(y^2+y'^2)-2e^{-\beta\hbar\omega}yy'}{1-e^{-2\beta\hbar\omega}}}.\end{aligned} \tag{S7}$$

The thermal density is then simply obtained by setting $y = y'$, which gives

$$\begin{aligned}\rho(y) &= e^{-\frac{\beta\hbar\omega}{2}} \frac{\alpha}{\sqrt{\pi}} \frac{1}{\sqrt{1-e^{-2\beta\hbar\omega}}} \cdot \\ &\cdot e^{-y^2\left[1+\frac{2e^{-\beta\hbar\omega}\left(e^{-\beta\hbar\omega}-1\right)}{1-e^{-2\beta\hbar\omega}}\right]}\end{aligned} \tag{S8}$$

after trivial rearrangements. As discussed in the main text, the thermal density is Gaussian with an ω-dependent normalization factor (first line of Equation S8) and a non-trivial scaling of the exponent. The latter will be rearranged to extract the quantum effective temperature from Equation 3 of the main text. Substituting $t = e^{-\beta\hbar\omega}$, we get

$$\begin{aligned}1 + \frac{2e^{-\beta\hbar\omega}\left(e^{-\beta\hbar\omega}-1\right)}{1-e^{-2\beta\hbar\omega}} &= 1 + \frac{2t(t-1)}{1-t^2} \\ = \frac{t^2-2t+1}{1-t^2} &= \frac{1-t}{1+t} = \frac{1-e^{-\beta\hbar\omega}}{1+e^{-\beta\hbar\omega}} \\ &= \tanh\left(\frac{\beta\hbar\omega}{2}\right),\end{aligned} \tag{S9}$$

[a)]Electronic mail: ondrej.marsalek@mff.cuni.cz

which gives

$$\rho(\Omega) \propto e^{-\frac{\omega}{\hbar}\tanh\left(\frac{\beta\hbar\omega}{2}\right)\Omega^2} \tag{S10}$$

for the thermal density in the original coordinates. Comparing the hyperbolic tangent factor with the form of a classical distribution ($\rho_{\mathrm{cl}}(y) \propto e^{-\beta V} = e^{-\frac{1}{2}\beta\omega^2\Omega^2}$) of the harmonic oscillator at a new inverse temperature β^*, we immediately get

$$\begin{aligned} \frac{\omega}{\hbar}\tanh\left(\frac{\beta\hbar\omega}{2}\right) &\equiv \frac{1}{2}\beta^*\omega^2 \\ \beta^*(\beta,\omega) &= \frac{2}{\hbar\omega}\tanh\left(\frac{\beta\hbar\omega}{2}\right), \end{aligned} \tag{S11}$$

as stated in the main text. Since the hyperbolic tangent approaches unity as its argument grows, it is always lower-valued than a linear function and thus always $\beta^* < \beta$. In turn, this means that the quantum harmonic oscillator is always at an effective temperature higher than the reference classical temperature and its density is thus always wider. Moreover, note that the classical limit $\beta^* \to \beta$ is reached as $\beta \to 0$ or $\omega \to 0$: at a given temperature, the higher frequency modes will have a more pronounced quantum effect and, in turn, at a given frequency, the quantum effect will be more pronounced at a lower temperature.

Density matching

An obvious technical problem with appending the thermal NMS at the minima at the MEP endpoints is the matching of the sampling density so that it is a smooth continuation of the linear MEP sampling (as indicated in Figure 1 of the main text). For practical purposes, one can achieve sufficient smoothness by separately choosing a linear density for the MEP sampling and a fixed number of points that are sampled thermally around the minima by a trial-and-error approach. However, this does not lead to an analytically smooth transition. That can be achieved by integrating the thermal Gaussian distributions at the minima over all dimensions but the one defined by the tangent vector to the MEP $\boldsymbol{\tau}$ at the minimum and finding a scaling factor that brings the density at the maximum of the Gaussian equal to the linear density ρ_0 of sampling along the MEP, yielding an analytic continuation. Rather inconveniently from a mathematical perspective, the MEP tangent $\boldsymbol{\tau}$ is, in general, not identifiable with any of the normal modes $\boldsymbol{\Omega}_i$, which diagonalize the Gaussian distribution. Therefore, to perform the integration over everything but the τ-direction, one needs to set up a new orthogonal basis built around this vector in which, however, the Gaussian is no longer diagonal. Still, the result of such integration will be a 1D Gaussian and its width will be given by the effective frequency $\widetilde{\omega}$

$$\rho(\tau) = \int \rho(\tau, \tau_2, \ldots, \tau_{3N})\, \mathrm{d}\tau_2 \ldots \mathrm{d}\tau_{3N} \propto e^{-\frac{1}{2}\beta\widetilde{\omega}^2\tau^2}. \tag{S12}$$

It can be shown (with the full proof being outside of the scope of the present work), that this effective frequency can be calculated from the following compact formula

$$\widetilde{\omega}^2 = \frac{\det(\mathbb{H})}{\det_{\tau,\tau}(\mathbb{H})}, \tag{S13}$$

where $\mathbb{H}$ is the Hessian matrix of the potential at the minimum and the symbol $\det_{\tau,\tau}$ denotes the minor of the Hessian matrix taken over the row and the column corresponding to the τ-dimension. Note that, unlike the full determinant, the minor is a basis-dependent operation and, therefore, the Hessian matrix must be transformed to the τ-oriented basis prior to the calculation of $\widetilde{\omega}$. The effective standard deviation of the 1D distribution is $\widetilde{\sigma} = 1/\sqrt{\beta\widetilde{\omega}^2}$. The aim at this point is to find a scaling factor α which ensures that the density at the configurational minimum $\alpha\rho(0)$ matches ρ_0. However, one cannot simply equate the two values directly, since the $\rho(\tau)$ comes from a mass-weighted coordinate system (note that the $\boldsymbol{\tau}$-based basis was obtained as a simple rotation of the normal mode basis, which itself is naturally mass-weighted) and ρ_0 is understood as a density along the non-weighted Cartesian MEP curve. Therefore, we need to calculate the effective mass μ of the τ-direction, which is the following weighted average

$$\mu = \frac{\boldsymbol{\tau}^T\mathbb{M}\boldsymbol{\tau}}{\boldsymbol{\tau}^T\boldsymbol{\tau}}, \tag{S14}$$

where $\mathbb{M}$ is the diagonal mass matrix. Then, we obtain the analytic expression

$$\alpha\rho(0) = \frac{\alpha}{\widetilde{\sigma}\sqrt{2\pi}} = \sqrt{\frac{1}{\mu}}\rho_0 \tag{S15}$$

which immediately gives the crucial density-matching condition

$$\alpha = \widetilde{\sigma}\sqrt{\frac{2\pi}{\mu}}\rho_0. \tag{S16}$$

The scaling parameter α thus captures how many times the original normalized density $\rho(\tau)$ needs to be increased in order to match at its peak the sampling density at the MEP. In practice, where the sampling of configurations is discrete and finite, one needs to sample the nearest-integer-to-α points to match the sampling density at the MEP.

S2. ADDITIONAL RESULTS

TTS of malonaldehyde proton transfer

The top panel of Figure S1 shows the distribution of the TTS geometries of malonaldehyde obtained at 300 K

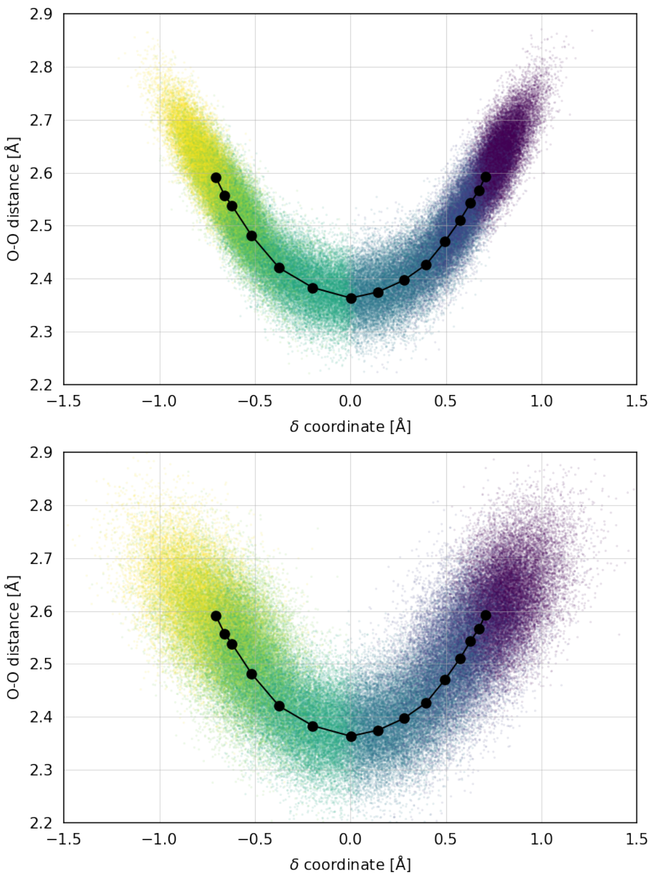

FIG. S1. Thermal geometries along the malonaldehyde proton-sharing MEP at 300 K generated using the classical formulation of TTS (top panel) and the quantum formulation of TTS that relies on β^* (bottom panel).

using the classical harmonic distributions in the mode directions perpendicular to the proton-sharing MEP. Three control points were selected for the TTS: the two symmetric minima at the edges of the MEP and the transition state at $\delta = 0$ Å. Each candidate geometry is colored by the assignment to its control point following the methodology described in Section II of the main text and illustrated in Figure 1 therein. The bottom panel of Figure S1 then shows the same situation but using the quantum formulation of TTS in which β^* is calculated for each degree of freedom. The use of the quantum densities leads to the characteristic broadening of the distribution of the geometries.

Path integral MD for malonaldehyde

The process described in the main paper for classical MD simulation can be done analogously for PIMD. TTS was used to generate structures along the proton-sharing MEP at an effective temperature of 300 K and 620 structures were selected during one QbC run for the initial model. Similar to the classical model, force disagreement during PIMD was low around the two free energy minima and along the MEP, but increased at the tails of the distribution, as can be seen in the left panel of Figure S2 To improve the MLP in these areas of the configuration space, two strategies were pursued. An iterative process, in which PIMD simulations and QbC processes with the structures of the PIMD trajectory as candidates are alternated until force disagreement is low along the whole PIMD trajectory. Or a single QbC run, in which TTS is used to create candidate structures along the MEP of the proton-transfer reaction as well as the MEP of the torsion of the C–C single bond of the propane back bone up until the transition state to include the strongly anharmonic vibrations in this direction of the configuration space. The distribution of the force disagreement for the second-generation MLP of the iterative process can be seen in the middle panel of Figure S2. Unlike the classical case in the main paper, a single additional PIMD–QbC sequence, which added 435 structures to the training data set, was sufficient to obtain an MLP capable of accurate PIMD simulations. In the right panel of Figure S2, the results from the extended TTS MLP can be seen. As for the classical case, the resulting model gives a low committee disagreement along the whole PIMD trajectory.

Using quantum models for classical MD

As the modes of harmonic oscillator are delocalized more strongly in the quantum than in the classical case, the structures generated using quantum TTS should cover a wider area of configuration space than those generated using classical TTS. While many of these structures are outside of the areas accessible during classical MD simulations, the areas that are accessible should still be covered well by the training data set selected from quantum structures and therefore, it should be possible to run classical MD with a quantum model. In Figure S3, the disagreements from classical MD simulations run with various models are shown. The models were trained either only on quantum structures, meaning structures originating from quantum TTS and PIMD simulations, or only on classical structures, meaning classical TTS and MD simulations. Particularly noteworthy are the differences between the two first-generation models of the iterative approach, which contain only structures from TTS along the proton-sharing MEP. Both models fail to accurately predict structures in the tails of the distribution. However, in this case, the quantum model fares better than the classical one in areas of high $d_{\mathrm{OO'}}$, which is likely a result of quantum TTS covering some areas not represented in the initial MEP due to the neglected torsion. For later models of the iterative approach as well as models from the extended TTS, this advantage of the quantum model vanishes and both models perform equally well. Similar to previous simulations, the extended TTS models have a slightly increased disagreement in the area of low $d_{\mathrm{OO'}}$, which is due to the

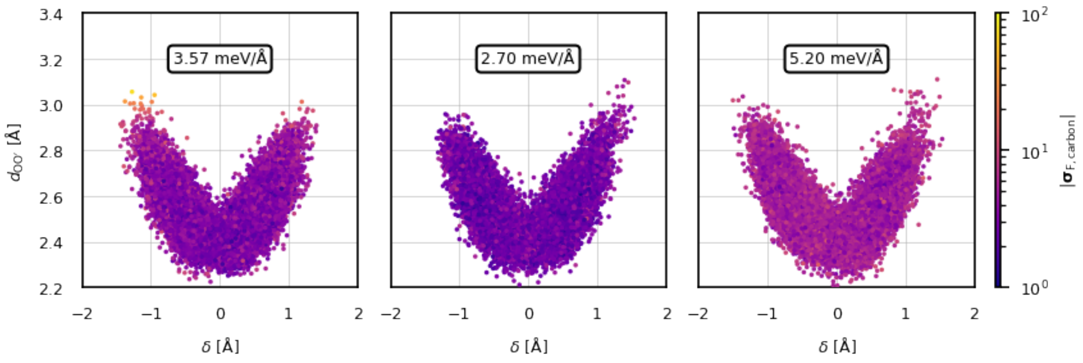

FIG. S2. The distribution of force disagreement of the carbon atoms along a PIMD trajectory for three different MLPs. The initial model trained on structures selected from the TTS candidate set (generated at an effective temperature of 300 K for path integral structures) is shown on the left and the model improved with structures selected from the PIMD trajectory of the initial model is shown in the middle panel. The model used to generate the data in the right panel was trained only on TTS structures, combining the contributions from the proton transfer MEP and the MEP of the torsion along the C–C single bond. The force disagreement is indicated by the color scale on the right and the mean disagreement in noted in each panel. While the middle and right panels exhibit an even distribution of disagreement, there is a sharp increase in disagreement at the tails of the distribution in the left panel. In all cases, only a subset of the complete PIMD trajectory is shown.

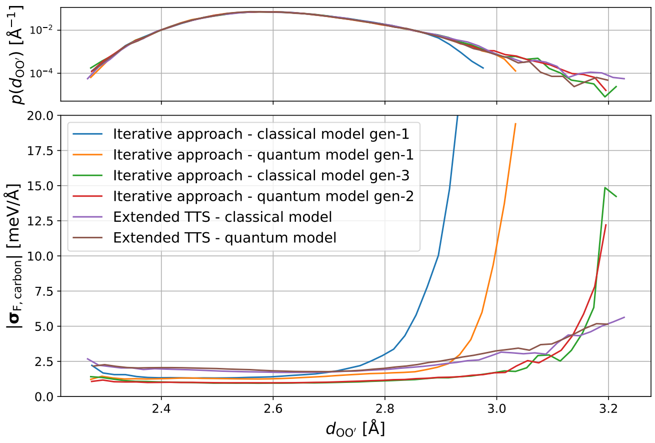

FIG. S3. The distribution of force committee disagreements from classical MD simulations run with different models. The bottom panel shows the disagreement for 6 different models, 3 of them are trained on classical structures and 3 are trained on path integral structures. The blue curve is cut off to improve visibility and would reach up to 40 meV/Å. The top panel shows the probability distribution along the $d_{OO'}$ coordinate of each trajectory on a logarithmic scale. Most simulations show a very similar distribution with the exception of the generation 1 models, whose trajectory does not reach regions of high $d_{OO'}$.

wider range of different structures present in these training data sets and therefore a less dense coverage of this central area. While using quantum models for classical simulations works well for malonaldehyde, there is no guarantee that it performs equally well for other systems, where this "dilution" of the training set due to broader coverage might result in a larger penalty in accuracy.

Validation of malonaldehyde MLPs

TABLE S1. Test root mean square errors for energies and forces. IA stands for iterative approach.

MLP	RMSE E [meV]	RMSE F [meV Å^{-1}]
IA generation 1	24.58	161.7
IA generation 2	1.89	18.7
IA generation 3	1.80	18.3
extended TTS	3.44	24.4

In the main text of the paper, the energy and force RMSEs on an independent test set for the final models were reported. The complete set of validation errors can be seen in table S1. While the later generations of the iterative approach and the extended TTS model performed well, the initial model performed poorly. However, the errors averaged over the whole test set tell only a part of the story, because they are not evenly distributed for all MLPs. As shown in Figure S4, the first generation model displays low force errors for most structures with a low d_{O-O}, which accounts for structures along the proton-sharing transition and the minima, but exceedingly large errors of up to 603 meV (energy) and 3908 meV Å^{-1} (forces) for $d_{O-O} > 2.8$. Due to the anharmonicity of the system, no structures from this region of the configuration space are included in the TTS along the proton sharing coordinate and hence such structures are absent from the training data set. Therefore, the model is not suitable for accurate MD simulations. The same effect can be observed in the second generation model, however less severe. The final models of both approaches, on the other hand, exhibit an even distribution of force RMSEs across the complete span of oxygen-oxygen distances.

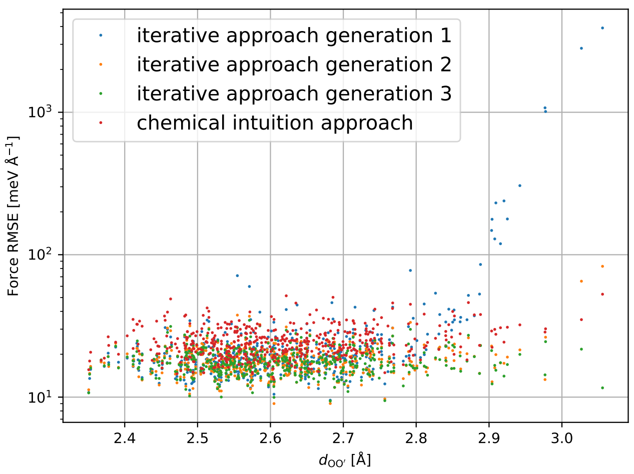

FIG. S4. Distribution of force RMSEs for the three generations of MLPs from the iterative approach and the MLP from the extended TTS approach along the distance between the two oxygen atoms. Of the 1000 structures of the test set, only the 500 structures sampled from an MD trajectory are shown here for clarity, as this subset includes the most insightful structures.

Overlap of umbrella windows in DABQDI

Figure S5 shows that the histograms of δ_1 values from the individual windows of the umbrella sampling simulation of the proton transfer in the DABQDI molecule exhibit sufficient overlap. Therefore, the combination of window spacing and harmonic restraint stiffness selected to obtain the results shown in Figure 7 of the main text represents an appropriate choice.

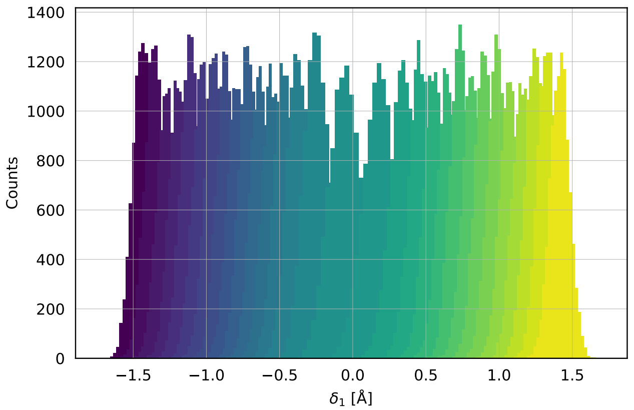

FIG. S5. Histograms of δ_1 values observed in each simulation window during the course of the umbrella sampling simulation of the DABQDI proton transfer reaction.

Error of the DABQDI proton-sharing free energy profile

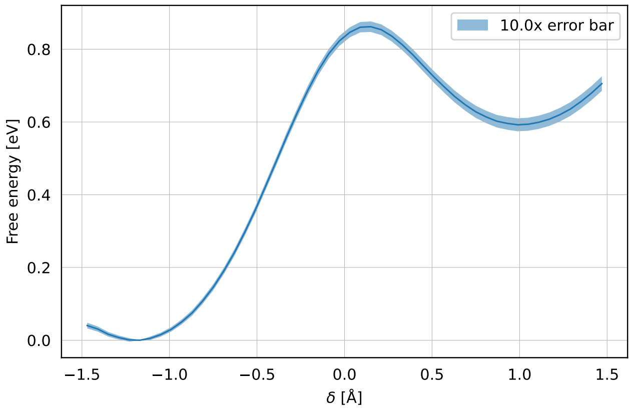

FIG. S6. The 10-fold multiple of the error in the estimated free energy relative to the global minimum is shown as light blue shading surrounding the mean free energy profile in dark blue (which displays the same data as shown in Figure 7 of the main text).

REFERENCES

[S1] F. G. Mehler, "Ueber die Entwicklung einer Function von beliebig vielen Variablen nach Laplaceschen Functionen höherer Ordnung," Journal für die reine und angewandte Mathematik **66**, 161–176 (1886).
